# CT-guided percutaneous marking of small pulmonary nodules with [^99m^Tc]Tc-Macrosalb is very accurate and allows minimally invasive lung-sparing resection: a single-centre quality control

**DOI:** 10.1007/s00259-023-06410-1

**Published:** 2023-08-31

**Authors:** Nikola Doncic, Christoph J. Zech, Damian Wild, Helga Bachmann, Makhmudbek Mallaev, Nikolay Tsvetkov, Aljaz Hojski, Martin T. L. Takes, Didier Lardinois

**Affiliations:** 1 Department of Thoracic Surgery, University Hospital Basel, Spitalstrasse 21, 4031 Basel, Switzerland; 2 Department of Radiology and Nuclear Medicine, Division of Interventional Radiology, University Hospital Basel, Basel, Switzerland; 3 Department of Radiology and Nuclear Medicine, Division of Nuclear Medicine, University Hospital Basel, Basel, Switzerland

**Keywords:** Pulmonary nodules, CT guided percutaneous marking, [^99m^Tc]Tc-Macrosalb, Video-assisted thoracoscopic surgery, Radio-guided surgery

## Abstract

**Purpose:**

The detection of small lung nodules in thoracoscopic procedure is difficult when the lesions are not located within the outer border of the lung. In the case of ground-glass opacities, it is often impossible to palpate the lesion. Marking lung nodules using a radiotracer is a known technique. We analysed the accuracy and safety of the technique and the potential benefits of operating in a hybrid operating room.

**Methods:**

57 patients, including 33 (58%) females with a median age of 67 years (range 21-82) were included. In 27 patients, we marked and resected the lesion in a hybrid room. In 30 patients, the lesion was marked at the department of radiology the day before resection. [^99m^Tc]Tc-Macrosalb (Pulmocis^®^) was used at an activity of 1 MBq in the hybrid room and at an activity of 3 MBq the day before to get technical feasible results. Radioactivity was detected using the Neoprobe^®^ detection system.

**Results:**

Precise detection and resection of the nodules was possible in 95% of the lesions and in 93% of the patients. Complete thoracoscopic resection was possible in 90% of the patients. Total conversion rate was 10%, but conversion due to failure of the marking of the nodule was observed in only 5% of the patients. Histology revealed 28 (37%) primary lung cancers, 24 (32%) metastases and 21 (28%) benign lesions. In 13 (23%) patients, minor complications were observed. None of them required additional interventions.

**Conclusion:**

The radio-guided detection of small pulmonary nodules is very accurate and safe after CT-guided injection of [^99m^Tc]Tc-Macrosalb. Performing the operation in a hybrid room has several logistic advantages and allows using lower technetium-99m activities. The technique allows minimally invasive lung sparing resection and prevents overtreatment of benign and metastatic lesions.

**Supplementary Information:**

The online version contains supplementary material available at 10.1007/s00259-023-06410-1.

## Introduction

The radiologic detection of small peripheral pulmonary lesions has increased, partly due to technical advances in chest CT, and mainly because lung imaging is performed much more frequently in screening and follow-up programs [1, 2]. A new classification of lung cancer has pointed out small ground-glass opacities that generally have a good prognosis [3–5]. The therapy of choice consists of the minimally invasive surgical resection, first for diagnostic, but eventually also for therapeutic reasons [6]. However, if the nodule is not located very close to the border of the lung, it is often impossible to localize by video-assisted thoracoscopic surgery (VATS).

The exact localization often requires an open approach, which is associated with potential discomfort and pain for the patient. Furthermore, it is sometimes nearly impossible to palpate and localize the lesion digitally in the case of ground-glass opacities. Finally, in the case of multiple nodules localized in different lung lobes, it is often difficult to palpate and precisely localize the nodules to be removed through the same mini-thoracotomy. It has been shown that two thirds of nodules with a diameter ≤10 mm, with a distance from the pleura >5 mm, or with non-solid attenuation are not localizable during VATS [7, 8].

The increased utilization of VATS has been correlated with increases in benign resection rates [9]. The IASLC recommends a 15% benign disease resection rate to maximize oncologic benefit while minimizing morbidity from nontherapeutic resections [10]. Therefore, it is also very important to precisely localize the nodule to be resected to avoid unnecessary loss of lung tissue during resection. Different methods have been developed to try to facilitate the localization of the nodule preoperatively: hookwires, dyes, radiotracer injected around the nodule bronchoscopically or percutaneously, coils, ultrasound-guided techniques – which are nowadays routine procedures. Advanced bronchoscopic techniques can often locate the nodule correctly, provided that the size is ≥10 mm, but the diagnostic yield still remains low (60-75%) [11].

At our department, we have introduced the technique of gamma-probe detection after preoperative transthoracic marking of the nodule with technetium-99m labelled albumin macro aggregates in 2019. We were interested in determining the accuracy of this method as a quality control measure in a consecutive series of patients.

## Material and methods

The project is registered by the Research Ethic Committee as a retrospective quality control, hence there is no statistical analysis of the gathered data. There is no special informed consent form, since we were applying the method already described in the literature for the given indication.

The patients were selected based on the size and the localization of the nodules: a) nodules that were deemed too small or too deep in the lung parenchyma for a classical VATS b) nodules too small for a realistic chance of a diagnostic CT-guided Tru-Cut biopsy, and c) nodules still amenable to a CT-guided percutaneous puncture by the interventional radiologist were chosen for the marking. Type c) were not linked to strict size- or distance-based criteria, but rather based on factors such as geometrical accessibility due to fissures and obstruction by the ribs or scapula. However, with experience we learnt that all those factors can be influenced by patient positioning. The choice of location to perform marking – hybrid operating room versus radiology ward – was based on the availability of the hybrid operating room during the coronavirus pandemic.

### Nodule marking

The marking was performed according to the standard technique for CT-guided pulmonary interventions at our department. In the hybrid operating room, the marking was performed under general anaesthesia with double-lumen intubation; in the radiology ward the day before surgery, it was done on an awake patient under local anaesthesia. As a first step we performed a planning CT without contrast in supine, prone or lateral position, according to the location of the nodule and at the discretion of the interventional radiologist. After planning, the patients were scrubbed and draped in a sterile fashion. The puncture was performed with a coaxial needle, ranging in size from 19G to 22G under CT-fluoroscopy in a “step and shoot” fashion. Once the position of the needle in the target lesion was confirmed, 1 ml [^99m^Tc]Tc-Macrosalb (Pulmocis^®^ kit containing 2 mg macrosalb for labelling with technetium-99m, Curium, France) was injected at an activity of 1 MBq directly prior to surgery and at an activity of 3 MBq the day before surgery. After the injection the needle was flushed with 0.5 ml sterile 0.9% NaCl and withdrawn. A final CT-fluoroscopy imaging was done to detect possible complications. Complications were collected from the already existing reports in the patient charts. Since the injected marking fluids might also be seen as ground-glass opacity around the nodule, small amounts of ground-glass around the lesion and minor complications of pneumothorax, for example, observed on same day of marking and resection were not counted as complications because the operation was performed immediately after marking. In general, we decided not to perform additional imaging verification of tracer deposition, such as a SPECT-CT scan, but rather assumed that the tracer would be in the region of the needle tip as documented with CT-fluoroscopy prior to injection.

### Nodule resection

**Fig. 1:**
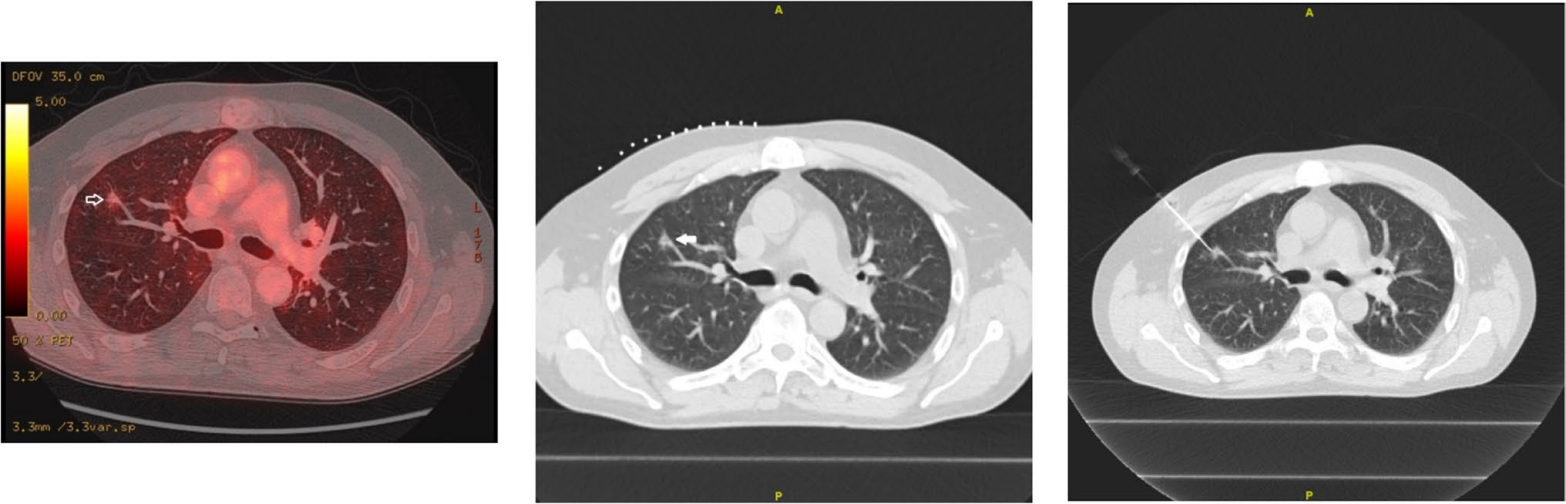
Nodule resection and example of primary adenocarcinoma. A 63-year-old patient with suspicious, faintly PET-positive, 8-mm ground-glass opacity. Histology revealed primary adenocarcinoma of the lung

**Fig. 2:**
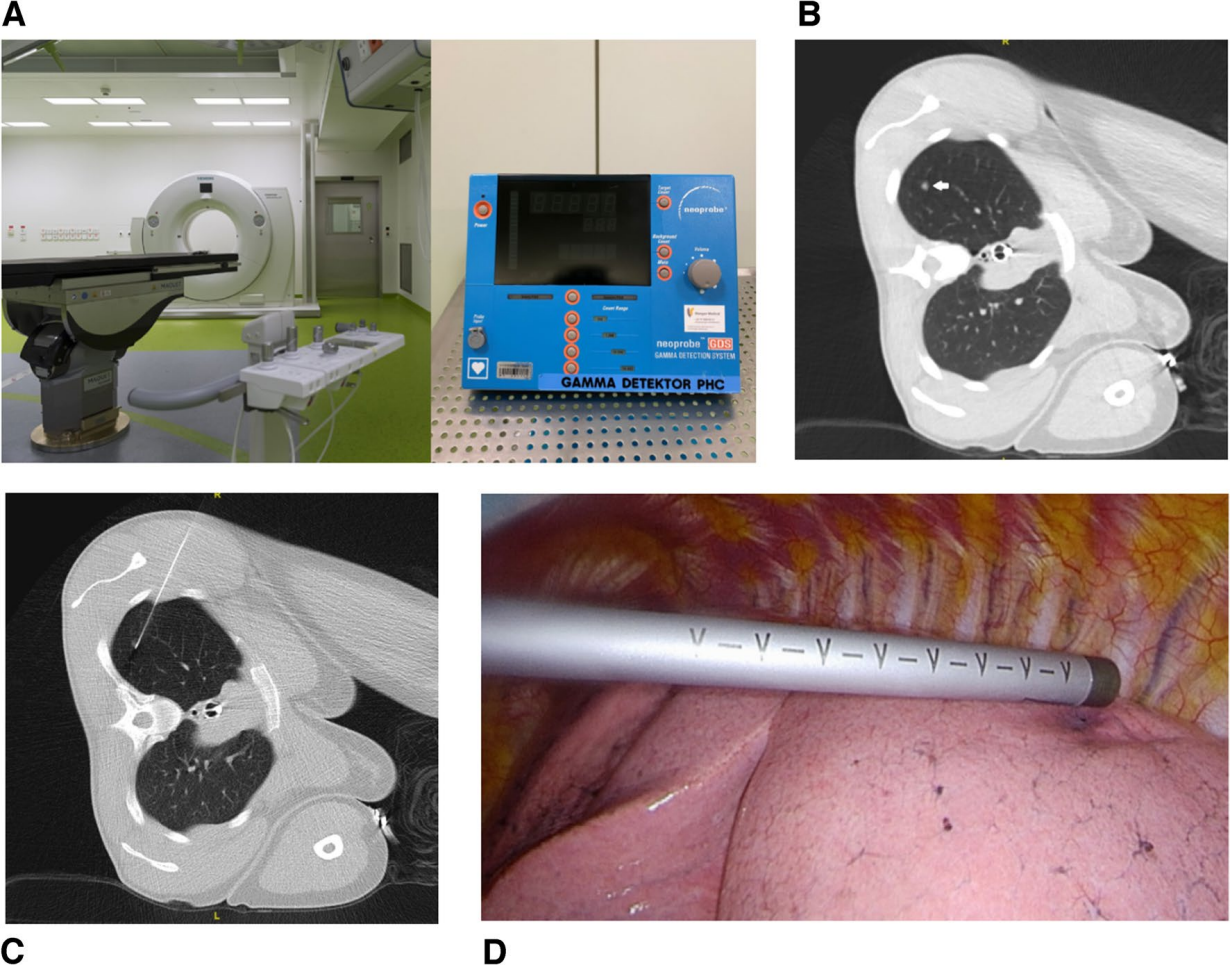
Nodule detection. **A** Hybrid operating room and Neoprobe console; **B** Planning CT scan of a 28-year-old patient with a history of clear cell sarcoma and suspected, then subsequently confirmed metastasis; **C** CT-guided puncture and nodule marking; **D** Intraoperative detection with the Neoprobe^®^ gamma detection system

Figure 1 gives an example of nodule resection and shows a primary adenocarcinoma. The resections were performed in a standard lateral position. After sterile scrubbing and draping, three incisions for the thoracoscopic ports were made according to the localization of the nodules. After extended adhesions had been ruled out, a 11 mm endoscopic detection probe of the Neoprobe^®^ gamma detection system (Devicor, USA) was introduced into the hemithorax to locate the radioactivity (Fig. 2). The detecting tip of the probe was slowly moved over the surface of the lung. It was then possible to precisely localize the point with the maximum activity with a high-pitched audio-signal and by the number of counts on the console. The identification of the area with the maximum activity was very accurate, as a small movement of 1-2 mm from this area was enough to register nearly no activity. The resections were performed as wedge resections using stapler devices. The specimen was removed from the chest cavity with an EndoBag and the maximum activity located and measured again in the specimen by the operating surgeon using the detection probe on a back table. The detection probe was also placed in the chest cavity to search for any remaining activity in the unresected lung. The specimen was then incised on the back table by the surgeon to determine if the nodule matched with the point of maximum activity. The specimen was sent to the pathologist for the frozen section. If the lesion was benign or revealed metastasis, the operation was stopped. If the nodule revealed non-small cell lung cancer, anatomical resection (segmentectomy or lobectomy with lymph node dissection) was performed under the same anaesthesia. If it was not possible to detect any activity with the detection probe, or if no nodule could be clearly identified in the specimen, a conversion to mini-thoracotomy was performed and the lung was palpated. Postoperative management was done according to the standards of the clinic.

## Results

Between April 2019 and December 2021, 57 patients, including 33 females (58%) and 24 males (42%), with a median age of 67 years (range 21-82) underwent the procedure. All patients were previously discussed at an interdisciplinary tumour board where the decision to obtain a histological confirmation was made based on nodule morphology, a change in size over time, a history of previous malignancy, or FDG-uptake on PET/CT imaging.

We performed marking and resections on 27/57 (47%) patients in the hybrid operating room with intraoperative CT on the same day directly prior to surgery, minimizing the time between marking and resection and providing the potential benefit of general anaesthesia for the CT-guided puncture. In 30/57 (53%) patients, the marking was performed in the radiology ward, followed by resection the day after in a standard operating room. Overall, 76 nodules were marked. In all patients whose lesion was deemed accessible for marking, the procedure was completed with the needle at the nodule, resulting in a technical success rate of 100%. Multiple nodules were marked in 14 patients, 2 nodules in 9 patients (16%) and 3 nodules in 5 patients (9%).

On average, the longest diameter of the lesions measured in preoperative CT imaging was 10 mm (SD 6.4, range 1-40) with a diameter of less than or equal to 5 mm in 18 lesions. The largest nodule was a ground-glass opacity 17 mm from the pleura. The mean distance between the nodule and pleura was 10.8 mm and (SD 8.1, range 1-39). The nodules were predominantly located in the upper lobes (Table 1). These parameters did not differ significantly between two cohorts: the mean of the longest diameter in the hybrid room was 9.4 mm (SD 5.3, range 1-22) and 10.5 mm (SD 7.3, range 2-40) in the radiology ward. The mean pleural distance was 10.9 mm (SD 8.5, range 1-27) in the hybrid room and 10.8 mm, (SD 7.7, range 1-39) in the radiology ward.

**Table 1:** Nodule characteristics and locations

*Localization of the marked nodules*		
Total number of marked nodules (n)	76	
	Hybrid room	Radiology ward
Right upper lobe [n (%)]	17 (47)	16 (40)
Right lower lobe	4 (11)	12 (30)
Middle lobe	1 (3)	0
Left upper lobe	9 (25)	7 (18)
Left lower lobe	5 (14)	5 (12)

In two patients with multiple marked nodules, two nodules (one in each patient) were not removed, as the final diagnosis of metastases was already clear after removing the other nodules and free-frozen analysis. In these cases, where the resection of the additional nodes would not have any prognostic and therapeutic benefit, we did not detect and resect these nodules and they were excluded from the analysis.

Correct detection of the nodules was successful in 70/74 (94.6%) lesions, respectively in 53/57 (93%) patients. We did not detect any activity with the probe in two patients, both marked and resected in the hybrid room. One 21 mm nodule was in the anterior segment of the right upper lobe, 10 mm from the pleura and near a lung fissure. The other was a 5 mm nodule in the posterior right upper lobe segment, 5 mm from the pleural surface. We were not able to clarify this as no image verification was available. We assumed that the tracer was most likely injected into the pleural space or there was a technical problem with the injection.

In the third patient, activity was easily detected at the surface of the lung. However, it was impossible to find any nodules in the specimen after wedge resection. The lesion corresponded to a small nodule of about 3 mm at a distance of 14 mm from the pleura in the right lower lobe on preoperative chest CT. In this patient, the procedure was converted to a mini-thoracotomy and the lung palpated. A large wedge resection was performed at the same location and where the nodule was found.

In the fourth patient, injection was performed around a ground-glass opacity (GGO). After locating the activity, a wedge resection was performed and a nodule removed, revealing a benign lesion. The GGO could still be identified at the 3-month follow-up chest CT. We believe that a small nodule close to the GGO was marked incorrectly, leading to the resection of the wrong lesion.

Table 2 gives the histology of the different nodules. It was important to note that 21 (28.4%) of the lesions were benign.

**Table 2:** Accuracy of the method and histology of nodules

	Number (%)	
*Accuracy of marked nodules detected*		
Total number of marked and removed nodules	74 (100)	
Nodules correctly detected with Neoprobe		70 (95)
Nodules not correctly detected with Neoprobe		4 (5)
*Histology of marked and resected nodules*		
Total number of primary lung cancer	28 (37.8)	
Adenocarcinoma		27
Squamous-cell carcinoma		1
Total number of metastases	24 (32.4)	
Adenocarcinoma of the lung		2
Adenocystic carcinoma of the tongue		1
Adrenocortical carcinoma		1
Cervical carcinoma		4
Chondrosarcoma		1
Clear-cell sarcoma		2
Colon and rectal carcinoma		7
Leiomyosarcoma		1
Malignant melanoma		1
Oropharynx carcinoma		1
Squamous-cell carcinoma of the lung		2
Thyroid cancer		1
Total number of benign nodules	21 (28.4)	
Adenomatous hyperplasia		2
Aspergilloma		1
Bronchiolar adenoma		1
Chronic inflammation		2
Drug-induced pneumopathy		3
Hamartoma		2
Intrapulmonary lymph nodes		4
Organizing pneumonia		4
Subpleural and interstitial scar		2
Total marked nodules not resected	1 (1.4)	

In 51/57 patients (89.5%), resection was successfully and completely performed with minimal invasion. In six patients (10.5%), conversion of the procedure was necessary. In the two patients without any detection of activity and in one patient without nodule in the specimen, a mini-thoracotomy and digital palpation of the nodules was required (Table 3). In two other patients, we had to convert despite successful detection of radioactivity, because the nodules were not amenable to minimally invasive resection. They were lying in posterior paravertebral parts of the lung (one ground-glass opacity, 15 mm in diameter and 5 mm away from the visceral pleura in the right lower lobe; and a 17 mm lesion, 11 mm away from the visceral pleura in left lower lobe). In one patient, conversion was due to severe adhesions, for a lesion in the right upper lobe, 9 mm in diameter and 8 mm away from the visceral pleura. This means that conversion was due to failed nodule marking in 3/57 patients (5.3%).

**Table 3:** Surgery data

*Operating room for marking the nodules*		
Hybrid [n (%)]	27 (47)	
Standard	30 (53)	
*Approach*		
Minimally invasive (VATS) [n (%)]	51 (90)	
Conversion to mini-thoracotomy/open	6 (10)	
	Hybrid room	Radiology ward
Failure to detect activity	2	
Anatomical position of the tumour (paravertebral)	1	1
Severe adhesions		1
No nodule in the resected specimen		1
Total	3	3

Although we did not systematically collect the data on the counting rates of the lesions, we did not observe large discrepancies in the rates for lesions marked in the hybrid room compared to those marked the previous day in the radiology ward and the timing of the marking did not seem to have any influence on the detectability. The number of counts itself was not so important, as we could accurately locate the activity with the detecting probe even in patients with a low count. We did not observe any relationship between nodule size and detectability. In total,18/76 (24%) nodules were sized 5 mm or smaller. Of the 18 lesions, only one (5.6%) could not be located correctly. Of the other lesions larger than 5 mm, two (3.6%) could not be located.

In 13/57 patients (22.8%), we observed post-interventional complications. The most common complication was small apical pneumothorax (8 patients), followed by minimal haemothorax (4) and vasovagal syncope (1). However, the highest grade of these complications according to the Clavien-Dindo classification [12] was 1. None of them required additional interventions. The complications all occurred in patients marked the day before the operation. They were considered potentially clinically significant because they could have led to additional interventions.

## Discussion

With the increased availability of CT diagnostic, follow-up and screening programs, the number of small peripherally detected nodules has considerably risen. Despite several guidelines and algorithms for classifying, follow-up and management of pulmonary nodules, it is often difficult to differentiate between benign and malignant nodules [13, 14]. Surgery very often offers the only possibility for histologically proving the benign or malignant character of the nodule. On the other hand, extended anatomical resection should be avoided whenever possible for benign lesions. In our series of patients, we found nearly 30% benign lesions [15–18], which is similar to some previous series. Additionally, metastases were also observed in about 30% of the nodules. Wedge resections are oncologically valuable procedures to resect metastases without the need for unnecessary segmentectomy. This finding emphasizes the need for the development of safe and feasible methods for precise localization of the nodules, allowing for lung tissue sparing resections.

There are several different methods of marking small pulmonary nodules, such as using different dyes, lipiodol with intraoperative fluoroscopy, coils, hookwires, bronchoscopic techniques. All of these methods, while reasonably feasible and accurate, are burdened with substantial failure rates and numerous complications, such as difficult visualization of dye in lungs with lots of anthracotic pigment, embolic events with non-soluble substances, dislodgement of hookwires and coils leading to higher rates of pneumothorax, haemorrhage and pain and ultimately the need for conversion to thoracotomy [19–24]. Also, marking with hookwires requires the resection of larger volumes of lung parenchyma, which should be avoided in case of benign and metastatic lesions [25]

Similar to previous animal and human studies, we found the detection of small pulmonary nodules with the gamma detection system to be very accurate and safe after CT-guided marking with [^99m^Tc]Tc-Macrosalb [15–18, 26]. The nodules were correctly localized in 95% of the nodules and in 93% of the patients. One big advantage of this method over other marking methods is that the volume for injection is low with less than 1 ml. Therefore, only a very thin needle is needed, which makes it possible to reach almost every spot of the lung and also address lesions below 5 mm. We used needle sizes from 19 to 22G. Furthermore, depending on personal preference, 20G Chiba needles seems to be the best compromise with regard to small access and few image artefacts versus stiffness to correct the needle path, even if the needle is already inserted a few centimetres (which is not possible with needles that are too thin). We refined the marking further in the setting of the hybrid operating room, where we observed several potential advantages for the patient, such as avoiding the discomfort of the percutaneous transthoracic injection by performing the [^99m^Tc]Tc-Macrosalb marking under general anaesthesia, reducing the risk of pneumothorax by performing the marking on an unventilated lung, shortening of the time between the [^99m^Tc]Tc-Macrosalb marking and the operation, and thus eliminating the need for placing the thoracic drainage in case of pneumothorax or haemothorax and in the use of a very low activity of technetium-99m.

Some researchers have used the lymphoscintigraphy or added iodine contrast to the radiotracer for further confirmation that the radiotracer is positioned correctly in the lung. We abstained from such measures. We considered the presence of the new ground-glass opacity around the targeted lesion after marking as sufficient confirmation. Furthermore, SPECT/CT imaging is not compatible with the hybrid room approach and would increase the complexity and cost of the procedure. Also, at least the CT part of SPECT/CT imaging would increase the patient’s radiation burden. In our series, there were only three cases where lymphoscintigraphy could have led to improved detection and successful resection, but at the expense of the additional time, resources and cost needed to transfer the patient to obtain additional imaging. As the detection rate of almost 95%, we believe confirmation of correct labelling is not necessary.

A possible addition to the technique is adding a hand-held gamma camera, which have been used previously to determine the resection margins [27]. Here we would use it to confirm the correct injection of radiotracer and increase the technique’s accuracy in the hybrid operating room. The addition of augmented-reality assistance could increase the success of the puncture and simultaneously reduce the time needed, the need for repeated CT-fluoroscopies and the amount of radiation that patients receive. Combining the radio-guided marking of the occult nodules with preoperative 3D modelling [28], which we have already implemented at our practice to plan anatomic segmentectomies, may also increase the probability of correct resection and help surgeons overcome a sometimes cumbersome mismatch between the position of the lesion intraoperatively in an unventilated lung and its position in the imaging and 3D models of the fully expanded lung.

The pneumothorax rate (8/30, 26.7%) in general was higher than expected from a sole puncture with a thin needle [29]. However, a drainage before surgery on the next day was not required in our cohort. This highlights the significant advantage of performing the procedure in a hybrid operating room, ruling out the potential discomfort if the pneumothorax increases in size overnight before the operation. The potential downside was the initially time-consuming coordination of the various disciplines involved in the procedure, which was additionally aggravated during the coronavirus pandemic and the time loss during repositioning of the patient after marking and prior to resection. This is attributable to the fact that the procedure was not yet completely standardized at the beginning in our particular setting, which increased the time spent under anaesthesia.

On the other hand, performing the procedure in a hybrid operating room has a lot of logistic advantages, as patients don’t need to be transferred to radiology the day before the operation, which is also time consuming and involves two separate interventions, as demonstrated by Batchala et al. [30] These two problems can be eliminated, or at least mitigated, by marking and resecting in a one-step procedure in a hybrid room. It also means the injection can be performed under general anaesthesia, which is more comfortable for the patient. We should also mention that, if the time for injection in the hybrid operating room might be considered like a prolongation of the time under general anaesthesia, the operation time itself should be shorter, since the technique allows precise localization and fast removal of the nodule. Unfortunately, it was impossible to confirm this in our series because the duration of the different steps of the procedure were not precisely collected. However, other authors came to the same conclusions [7]. It was also impossible to analyse if the injection time was shorter when performing the puncture on an unventilated, stationary lung and with increasing experience.

With time, we could further reduce radiation burden without compromising detection by lowering the activity of the radioactive tracer from 10 to 3 MBq when marking was performed the day before resection and from 3 to 1 MBq when it was done on the same day, which is several times less than in recently published series [25, 30, 31]. Since such low activities of technetium-99m do not pose any known risks and side effects for the patient, the operating personnel and the pathologist, no additional safety measures needed to be implemented.

We could perform minimally invasive lung tissue sparing resections in the great majority of the patients. These limited wedge resections were able to provide us the definitive precise histological diagnosis and allowed us to avoid overtreatment of benign lesions by anatomical resection. The procedures were safe and without any complications requiring additional medical interventions. Another great advantage of this method is the possibility of real-time control of the exact location of the nodule with the gamma detection probe. The system is very selective, as very small rotation movements of the probe are enough to show a big difference in the counts. This allows for precise definition of the resection margins.

Previous papers described the use of the CT-guided percutaneous marking of nodules with certain limitations of the technique. Ambrogi et al. [15] and Galetta et al. [26] reported diffusion of radiotracer in emphysematous lungs, which led to a wider area of radioactivity and reduced resection accuracy. In our series of patients, we did not find any nodules in the resected lung in one patient. However, the lung was not particularly emphysematous, and we could locate the activity accurately. Other authors [17, 25, 26, 30, 31] mentioned the possibility of spillage of the tracer in the pleural space. We encountered this situation in two patients. It was impossible to find any activity with the detecting probe. In one patient, the nodule was localized close to a lung fissure, which could explain the extravasation of the tracer during injection.

In summary, the radio-guided detection of small pulmonary nodules after CT-guided injection of [^99m^Tc]Tc-Macrosalb has been proven to be very accurate and safe in our patient series. Performing the operation in a hybrid room resulted in several potential advantages for the patient and allowed the use of lower technetium-99m activities. The technique allowed minimally invasive lung sparing resection, avoiding overtreatment of benign lesions and metastasis.

### Supplementary Information

Below is the link to the electronic supplementary material.

Supplementary file1 (MP4 49314 KB)

## Data Availability

The datasets generated and analysed during the current project are available from the corresponding author upon reasonable request.

## Abbreviations

CT: Computed tomography
FDG: Fluorodeoxyglucose
G: Gauge
GGO: Ground-glass opacity
IASLC: International Association for the Study of Lung Cancer
MBq: Megabecquerel
mg: Milligram
ml: Millilitre
mm: Millimetre
n: Number
%: Percent
PET: Positron emission tomography
SPECT: Single photon emission computed tomography
NaCl: Sodium chloride
SD: Standard deviation
[^99m^Tc]: Technetium-99m
VATS: Video-assisted thoracoscopic surgery

## References

[1] de Koning HJ, van der Aalst CM, de Jong PA, Scholten ET, Nackaerts K, Heuvelmans MA, et al. Reduced lung-cancer mortality with volume CT screening in a randomized trial. N Engl J Med. 2020;382:503–13. 31995683 10.1056/NEJMoa1911793

[2] Becker N, Motsch E, Trotter A, Heussel CP, Dienemann H, Schnabel PA, et al. Lung cancer mortality reduction by LDCT screening-results from the randomized German LUSI trial. Int J Cancer. 2020;146:1503–13. 31162856 10.1002/ijc.32486

[3] Wang C, Wu Y, Li J, Ren P, Gou Y, Shao J, et al. Distinct clinicopathologic factors and prognosis based on the presence of ground-glass opacity components in patients with resected stage I non-small cell lung cancer. Ann Transl Med. 2020;8:1133. 33240982 10.21037/atm-20-4971 PMC7576059

[4] Kagimoto A, Tsutani Y, Handa Y, Mimae T, Miyata Y, Okada M. Clinical features and prognosis of clinical N0 non-small cell lung cancer exceeding 30 mm. Jpn J Clin Oncol. 2020;50:1306–12. 32901276 10.1093/jjco/hyaa167

[5] Handa Y, Tsutani Y, Okada M. Transition of treatment for ground glass opacity-dominant non-small cell lung cancer. Front Oncol. 2021;11:655651. 33937064 10.3389/fonc.2021.655651 PMC8082027

[6] Lim E, Batchelor Tim JP, Dunning J, Shackcloth M, Anikin V, Naidu B, et al. Video-assisted thoracoscopic or open lobectomy in early-stage lung cancer. NEJM Evidence. 2022;1:EVIDoa2100016. 10.1056/EVIDoa2100016 38319202

[7] Taton O, Sokolow Y, Bondue B, Vandermeeren C, Kuylen MV, Gevenois PA, et al. Cryobiopsy and dye marking guided by electromagnetic navigation bronchoscopy before resection of pulmonary nodule. Respir Med Res. 2022;81:100911. 35468469 10.1016/j.resmer.2022.100911

[8] Suzuki K, Nagai K, Yoshida J, Ohmatsu H, Takahashi K, Nishimura M, et al. Video-assisted thoracoscopic surgery for small indeterminate pulmonary nodules: indications for preoperative marking. Chest. 1999;115:563–8. 10027460 10.1378/chest.115.2.563

[9] Kuo E, Bharat A, Bontumasi N, Sanchez C, Zoole JB, Patterson GA, et al. Impact of video-assisted thoracoscopic surgery on benign resections for solitary pulmonary nodules. Ann Thorac Surg. 2012;93:266-72; discussion 72-3. 10.1016/j.athoracsur.2011.08.035 22075217

[10] Field JK, Smith RA, Aberle DR, Oudkerk M, Baldwin DR, Yankelevitz D, et al. International Association for the Study of Lung Cancer Computed Tomography Screening Workshop 2011 report. J Thorac Oncol. 2012;7:10–9. 22173661 10.1097/JTO.0b013e31823c58ab

[11] Sainz Zuñiga PV, Vakil E, Molina S, Bassett RL Jr, Ost DE. Sensitivity of radial endobronchial ultrasound-guided bronchoscopy for lung cancer in patients with peripheral pulmonary lesions: an updated meta-analysis. Chest. 2020;157:994–1011. 31738928 10.1016/j.chest.2019.10.042

[12] Dindo D, Demartines N, Clavien PA. Classification of surgical complications: a new proposal with evaluation in a cohort of 6336 patients and results of a survey. Ann Surg. 2004;240:205–13. 15273542 10.1097/01.sla.0000133083.54934.ae PMC1360123

[13] MacMahon H, Naidich DP, Goo JM, Lee KS, Leung ANC, Mayo JR, et al. Guidelines for management of incidental pulmonary nodules detected on CT images: from the Fleischner Society 2017. Radiology. 2017;284:228–43. 28240562 10.1148/radiol.2017161659

[14] Detterbeck FC, Lewis SZ, Diekemper R, Addrizzo-Harris D, Alberts WM. Executive summary: diagnosis and management of lung cancer, 3rd ed: American College of Chest Physicians evidence-based clinical practice guidelines. Chest. 2013;143:7s–37s. 23649434 10.1378/chest.12-2377

[15] Ambrogi MC, Melfi F, Zirafa C, Lucchi M, De Liperi A, Mariani G, et al. Radio-guided thoracoscopic surgery (RGTS) of small pulmonary nodules. Surg Endosc. 2012;26:914–9. 22011947 10.1007/s00464-011-1967-8

[16] Chella A, Lucchi M, Ambrogi MC, Menconi G, Melfi FM, Gonfiotti A, et al. A pilot study of the role of TC-99 radionuclide in localization of pulmonary nodular lesions for thoracoscopic resection. Eur J Cardiothorac Surg. 2000;18:17–21. 10869935 10.1016/S1010-7940(00)00411-5

[17] Grogan EL, Jones DR, Kozower BD, Simmons WD, Daniel TM. Identification of small lung nodules: technique of radiotracer-guided thoracoscopic biopsy. Ann Thorac Surg. 2008;85:S772-7. 18222215 10.1016/j.athoracsur.2007.10.105

[18] Bellomi M, Veronesi G, Trifirò G, Brambilla S, Bonello L, Preda L, et al. Computed tomography-guided preoperative radiotracer localization of nonpalpable lung nodules. Ann Thorac Surg. 2010;90:1759–64. 21095303 10.1016/j.athoracsur.2010.08.016

[19] Gonfiotti A, Davini F, Vaggelli L, De Francisci A, Caldarella A, Gigli PM, et al. Thoracoscopic localization techniques for patients with solitary pulmonary nodule: hookwire versus radio-guided surgery. Eur J Cardiothorac Surg. 2007;32:843–7. 17913505 10.1016/j.ejcts.2007.09.002

[20] Ciriaco P, Negri G, Puglisi A, Nicoletti R, Del Maschio A, Zannini P. Video-assisted thoracoscopic surgery for pulmonary nodules: rationale for preoperative computed tomography-guided hookwire localization. Eur J Cardiothorac Surg. 2004;25:429–33. 15019673 10.1016/j.ejcts.2003.11.036

[21] Mayo JR, Clifton JC, Powell TI, English JC, Evans KG, Yee J, et al. Lung nodules: CT-guided placement of microcoils to direct video-assisted thoracoscopic surgical resection. Radiology. 2009;250:576–85. 19188326 10.1148/radiol.2502080442

[22] Vandoni RE, Cuttat JF, Wicky S, Suter M. CT-guided methylene-blue labelling before thoracoscopic resection of pulmonary nodules. Eur J Cardiothorac Surg. 1998;14:265–70. 9761435 10.1016/S1010-7940(98)00160-2

[23] Watanabe K, Nomori H, Ohtsuka T, Kaji M, Naruke T, Suemasu K. Usefulness and complications of computed tomography-guided lipiodol marking for fluoroscopy-assisted thoracoscopic resection of small pulmonary nodules: experience with 174 nodules. J Thorac Cardiovasc Surg. 2006;132:320–4. 16872957 10.1016/j.jtcvs.2006.04.012

[24] Moon SW, Wang YP, Jo KH, Kwack MS, Kim SW, Kwon OK, et al. Fluoroscopy-aided thoracoscopic resection of pulmonary nodule localized with contrast media. Ann Thorac Surg. 1999;68:1815–20. 10585064 10.1016/S0003-4975(99)00764-X

[25] Vollmer I, Páez-Carpio A, Sánchez-Lorente D, Boada M, Martínez D, Sánchez M, et al. Preoperative localization of lung nodules: a comparative analysis of hookwire and radio-guided procedures. J Thorac Dis. 2022;14:4329–40. 36524098 10.21037/jtd-22-552 PMC9745529

[26] Galetta D, Rampinelli C, Funicelli L, Casiraghi M, Grana C, Bellomi M, et al. Computed tomography-guided percutaneous radiotracer localization and resection of indistinct/small pulmonary lesions. Ann Thorac Surg. 2019;108:852–8. 31075251 10.1016/j.athoracsur.2019.03.102

[27] Vollmer I, Sánchez-Izquierdo N, Martínez D, Sánchez-Lorente D, Casanueva-Eliceiry S, Boada M, et al. Role of a portable gamma-camera with optical view for margins assessment of pulmonary nodules resected by radioguided surgery. Eur J Nucl Med Mol Imaging. 2021;49:361–70. 34185137 10.1007/s00259-021-05466-1

[28] Sadeghi AH, Mathari SE, Abjigitova D, Maat A, Taverne Y, Bogers A, et al. Current and future applications of virtual, augmented, and mixed reality in cardiothoracic surgery. Ann Thorac Surg. 2022;113:681–91. 33347848 10.1016/j.athoracsur.2020.11.030

[29] Pradella M, Trumm C, Stieltjes B, Boll DT, Zech CJ, Huegli RW. Impact factors for safety, success, duration and radiation exposure in CT-guided interventions. Br J Radiol. 2019;92:20180937. 31045438 10.1259/bjr.20180937 PMC6636272

[30] Batchala PP, Mathew PF, Martin LW, Wankhar B, Ojili V, Nepal P, et al. CT guided injection of (99m)Tc-MAA for lung nodule localization prior to VATS. Clin Imaging. 2022;91:97–104. 36057205 10.1016/j.clinimag.2022.08.016

[31] Manca G, Davini F, Tardelli E, De Liperi A, Falaschi F, Melfi F, et al. Clinical impact of radioguided localization in the treatment of solitary pulmonary nodule: a 20-year retrospective analysis. Clin Nucl Med. 2018;43:317–22. 29432343 10.1097/RLU.0000000000001997

